# Interplay between Myokine Profile and Glycemic Control in Type 2 Diabetes Mellitus Patients with Heart Failure

**DOI:** 10.3390/diagnostics12122940

**Published:** 2022-11-24

**Authors:** Alexander A. Berezin, Zeljko Obradovic, Evgen V. Novikov, Elke Boxhammer, Michael Lichtenauer, Alexander E. Berezin

**Affiliations:** 1 Zaporozhye Medical Academy of Postgraduate Education, 20, Vinter Av., 69096 Zaporozhye, Ukraine; 2 Klinik Barmelweid, Department of Psychosomatic Medicine and Psychotherapy, 5017 Barmelweid, Switzerland; 3 Department of Cardiology, Vita Center, 69000 Zaporozhye, Ukraine; 4 Department of Propedeutics of Internal Medicine, Radiology and Radiation Therapy, Zaporozhye State Medical University, 26, Mayakovsky Av., 69035 Zaporozhye, Ukraine; 5 Department of Internal Medicine II, Division of Cardiology, Paracelsus Medical University Salzburg, 5020 Salzburg, Austria; 6 Department of Internal Medicine, Zaporozhye State Medical University, 26, Mayakovsky Av., 69035 Zaporozhye, Ukraine

**Keywords:** type 2 diabetes mellitus, heart failure, myokines, irisin, apelin, myostatin, adropin, biomarkers, prediction

## Abstract

Type 2 diabetes mellitus (T2DM) remains a powerful predictor of progressive heart failure (HF), but it is not clear whether altered glycemic control interferes with HF progression via an impaired profile of circulating myokines. The aim was to investigate plausible effects of glucose control on a myokine signature in T2DM patients affected by chronic HF. We selected 372 T2DM patients from the local database and finally included 314 individuals suffering from chronic HF and subdivided them into two groups according to glycosylated hemoglobin (HbA1c) levels (<6.9% and ≥7.0%). Echocardiography and Doppler examinations along with biomarker measurements were performed at the baseline of the study. The results showed that irisin levels were significantly lower in patients with HbA1c ≥ 7.0% than in those with HbAc1 < 6.9%, whereas concentrations of apelin, myostatin and adropin did not significantly differ between these two groups. We also identified numerous predictors of poor glycemic control, but only N-terminal brain natriuretic propeptide (odds ratio [OR] = 1.07; 95% confidence interval [CI] = 1.02–1.10, *p* = 0.04) and irisin (OR = 1.09; 95% CI = 1.04–1.17, *p* = 0.001) remained independent predictors of the dependent variable. In conclusion, we found that decreased levels of irisin were associated with poor glycemic control in T2DM patients with HF regardless of clinical conditions and other biomarkers.

## 1. Introduction

Heart failure (HF) remains a leading cause of in-hospital mortality and readmission of patients with established cardiovascular disease (CVD) worldwide [1]. Although the total number of new-onset cases of HF with reduced ejection fraction (HFrEF) has remained stable over the past decade, especially in developed countries, there seems to be strong relationship between the steady increase in HF prevalence worldwide and that the prevalence of HF with preserved ejection fraction (HFpEF) [2,3]. Moreover, the prevalence of HFpEF is not only rising in the general and hospital populations, but it is also currently significantly higher than that of HFrEF [4]. HF results from age, gender, ischemic/non-ischemic etiology, a profile of comorbidities including type 2 diabetes mellitus (T2DM), atrial fibrillation, anemia, and is also impacted by conventional CV risk factors, such hypertension, obesity, and dyslipidemia [5,6].

T2DM is highly prevalent in HF patients, and poor glycemic control is strongly associated with poor clinical outcomes, regardless of the HF phenotype [7]. Among T2DM patients, HFpEF is considered to be the most common disease phenotype, and conventional predictive biomarkers such as natriuretic peptides (NPs) were found not to be accurately associated with prognosis, unlike in HFrEF [8]. In contrast to NPs, some myokines produced by skeletal muscles and myocardium, mainly irisin, apelin and adropin, are associated with metabolic abnormalities including hyperglycemia, insulin resistance and skeletal muscle metabolism, adipose tissue browning in T2DM with adverse cardiac remodeling, skeletal muscle weakness/myopathy and finally HFpEF [9]. Previous studies reported that both T2DM patients and HF patients had lower concentrations of irisin than do healthy individuals, whereas data on the alteration of circulating levels of apelin, myostatin and adropin in chronic HF patients depending on the presence of T2DM remain controversial [10,11,12,13]. However, there is evidence of the fact that irisin and apelin have yielded prognostic values for HFpEF regardless of NPs [14,15]. It is still uncertain whether altered glycemic control in T2DM patients with chronic HF corresponds to a difference in circulating levels of these myokines and whether the myokine profile should be considered as a marker of advanced disease. The aim of this study was to explore the plausible impact of glucose control on the myokine signature in T2DM patients affected by chronic HF.

## 2. Materials and Methods

### 2.1. Study Design and Cohorts of Participants

**Figure 1:**
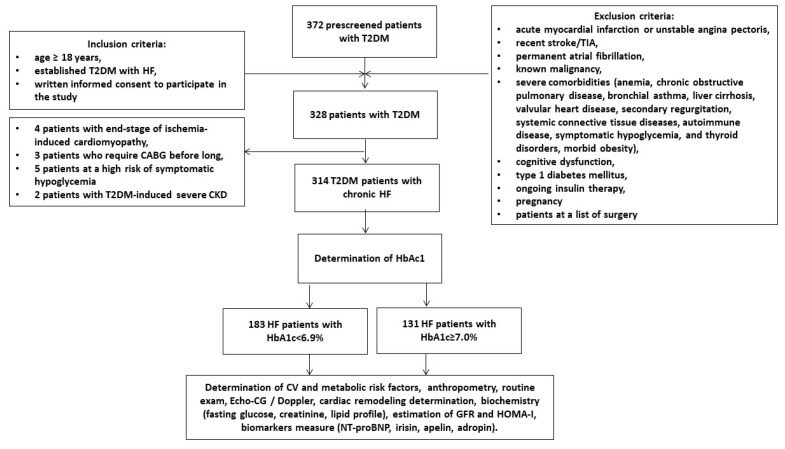
Flow chart of the study design. Abbreviations: CABG, coronary artery bypass grafting; CV, cardiovascular; CKD, chronic kidney disease; GFR, glomerular filtration rate; HF, heart failure; HbA1c, glycated hemoglobin; HOMA-IR, Homeostatic Assessment Model of Insulin Resistance; N-terminal brain natriuretic pro-peptide; T2DM, type 2 diabetes mellitus; TIA, transient ischemic attack.

A total of 372 patients with T2DM from the local database of the Vita-Centre private hospital (Zaporozhye, Ukraine) were prescreened and then invited to participate in the study. We used any medical records, discharge reports, laboratory reports, and communications with general practitioners to qualify the patients as candidates for participating in the study according to inclusion/exclusion criteria (Figure 1). The inclusion criteria were age ≥ 18 years, T2DM, established HF, and written consent to participate in the study. Exclusion criteria were acute coronary syndrome/myocardial infarction or unstable angina pectoris, recent stroke/transient ischemic attack, known malignancy, severe co-morbidities (anemia, chronic lung and liver diseases, known inherited and acquired heart defects, symptomatic severe hypoglycemia, morbid obesity, systemic connective tissue diseases, autoimmune disease, cognitive dysfunction and thyroid disorders), type 1 diabetes mellitus, ongoing insulin therapy and pregnancy. We ultimately enrolled 314 individuals with T2DM who had chronic HF and subdivided them into two groups depending on their HbA1c levels (<6.9% and ≥7.0%).

### 2.2. Determination of Co-Morbidities and Measurement of Anthropometric Parameters

We used the conventional clinical guidelines of the European Society of Cardiology (ESC) [16,17,18,19,20,21] to determine co-morbidities and risk factors including hypertension, dyslipidemia, T2DM, coronary artery disease, chronic kidney disease and HF. Poor glycemic control was defined as HbA1c ≥ 7.0% [16]. Height, weight, waist circumference, hip-to-waist ratio (WHR) and body mass index (BMI) were determined according to current recommendations [22]. Microalbuminuria was defined as an abnormally increased urinary albumin excretion rate in the range of 30–299 mg/g creatinine [23].

### 2.3. Examination of Hemodynamics

Blinded ultra-sonographers performed B-mode echocardiography and Doppler examination of the patients with the Vivid T8 diagnostic system (“GE Medical Systems”, Freiburg, Germany) in compliance with current guidelines [24]. The left ventricular ejection fraction (LVEF) was measured by the Simpson method. Left ventricular end-diastolic (LVEDV) and end-systolic (LVESV) volumes as well as the left atrial volume (LAV) were sampled in the apical 4-chamber view and the LAV index (LAVI) was calculated as the ratio of LAV to body surface area (BSA). Measures of diastolic performance include early diastolic blood filling (E), the longitudinal strain ratio (e’) and the ratio of these two (E/e’). The estimated E/e’ ratio was expressed as the ratio equation of E wave velocity to the averaged medial and lateral e’ velocity [24]. Left ventricular (LV) hypertrophy (LVH) was detected according to conventional echocardiographic criteria, which included LV myocardial mass index (LVMMI) ≥ 125 g/m^2^ or ≥ 110 g/m^2^ in males and females, respectively [24].

### 2.4. Diet and Medications

All T2DM patients enrolled in the study received a personally developed lifestyle modification program and dietary recommendations along with an adjusted dose of metformin and/or sodium-glucose cotransporter-2 (SGLT2) inhibitor. Other concomitant medications included renin–angiotensin–aldosterone blockers (angiotensin-converting enzyme inhibitors [ACEIs]/angiotensin receptor blockers [ARBs]; sacubitril-valsartan sodium, an angiotensin receptor/neprilysin inhibitor [ARNI]; and mineralocorticoid receptor antagonists [MRAs]) and beta-blockers. Loop diuretics, the i/f blocker ivabradine, lipid-lowering agents and antiplatelet drugs were prescribed when needed.

### 2.5. Blood Sampling, Storage and Measurement of Biomarkers

Fasting blood samples from patients were collected from an antecubital vein (3–5 mL) and maintained at 4 °C. After centrifugation (3000 r/min, 30 min) the collected serum aliquots were immediately stored at ≤−70 °C until analysis. Serum concentrations of NT-proBNP, irisin, apelin, myostatin and adropin were determined using commercially available enzyme-linked immunosorbent assay (ELISA) kits (Elabscience, Houston, TX, USA) according to the manufacturer’s instructions. All ELISA data were analyzed according to the standard curve and each sample was measured in duplicate, and the mean value was finally analyzed. Both intra- and inter-assay coefficients of variability for each biomarker were <10%.

Conventional biochemistry parameters were routinely measured at Vita-Centre, the local biochemical laboratory (Zaporozhye, Ukraine), using a Roche P800 analyzer (Basel, Switzerland). We used the CKD-EPI formula to estimate the glomerular filtration rate (GFR) [25]. Insulin resistance was evaluated using the Homeostatic Assessment Model of Insulin Resistance (HOMA-IR) [26].

### 2.6. Statistics

Statistical analysis was performed using SPSS 11.0 for Windows and GraphPad Prism v9 (GraphPad Software, San Diego, CA, USA). Continuous variables were expressed as means ± SD for parametric data, and we reported the median or interquartile range [IQR] according to whether they were normally distributed or not. The Kolmogorov–Smirnov test was used to test for a normal distribution. The distribution of dichotomous values was analyzed with a Chi-square test. We performed one-way analysis of variance (ANOVA) and the Tukey test for comparisons of variables between groups of patients with HbA1c < 6.9% and HbA1c ≥ 7.0%, and we used the Spearman distribution for correlations. Predictors of poor glycemic control were determined by univariate and multivariate linear regression analysis. We reported odds ratios (ORs) and 95% confidence intervals (95% CIs) for each variable included in the regression analysis. The predictive value of NT-proBNP and irisin for poor glycemic control was reclassified using integrated discrimination indices (IDI) and net reclassification improvement (NRI). Differences were considered significant at the *p* < 0.05 level of statistical significance.

## 3. Results

### 3.1. General Characteristics of the Patients

The entire population of the patients was mainly composed of men (65.3%) aged 40 to 62 years (Table 1). The majority of them had cardiovascular risk factors, such as dyslipidemia (85.3%), hypertension (85.0%), obesity (50.3%) and smoking (50.0%), as well as CVD including stable coronary artery disease (43.0%), paroxysmal/persistent forms of atrial fibrillation (22.9%), LVH (84.7%) and microalbuminuria (34.7%). With respect to the BMI, waist circumference and WHR, the averages were 26.1 kg/m^2^, 86.5 cm and 0.86 units, respectively.

The proportion of patients with HFpEF, HFmrEF and HFrEF was 39.5%, 33.4% and 27.1%, respectively. In addition, 46 (14.6%), 165 (52.5%) and 103 (32.8%) patients from the entire group had an HF-NYHA functional class of I, II and III, respectively. The mean LVEF was 49%, the mean LVMMI was 164 g/m^2^ and the mean LAVI was 46 mL/m^2^. Amongst the biochemical parameters, the average NT-proBNP level was 2855 pmol/mL, the average fasting glucose concentration was 6.65 mmol/L and the average HbA1c value was 7.01%. All patients received conventional therapies for HF, T2DM and comorbidities. We did not find significant differences between cohorts in terms of age, gender, anthropomorphic parameters, the presentation of dyslipidemia, hypertension, coronary artery disease, smoking and HF phenotypes, nor in terms of systolic and diastolic blood pressure, LVEDV, LVESV, LVEF, eGFR, NT-proBNP levels, HOMA-IR scores, creatinine levels, lipid profiles and concomitant medications. In contrast, T2DM patients with HbA1c ≥ 7.0% were more likely to have paroxysmal/persistent forms of atrial fibrillation (AF), abdominal obesity, microalbuminuria and LVH than patients with HbAc1< 6.9%. However, individuals with HbA1c ≥ 7.0% demonstrated significantly higher values of LVMMI, LAVI, E/e’ and concentrations of fasting glucose than those who had HbA1c < 6.9%.

### 3.2. Circulating Levels of Myokines in the Patients Included in the Study

**Figure 2:**
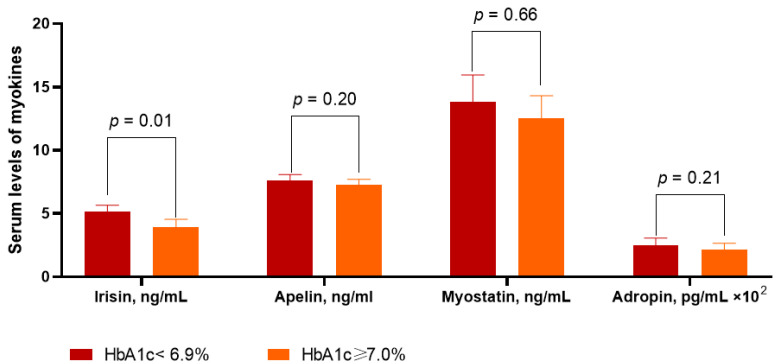
Myokine levels in T2DM patients with HF depending on glucose control. Abbreviations: HbA1c, glycosylated hemoglobin.

Levels of irisin were significantly higher in patients with HbA1c < 6.9% than in those with HbA1c ≥ 7.0% (5.12 ng/mL; 95% CI = 3.90–7.66 ng/mL vs. 3.90 ng/mL; 95% CI = 3.20–5.25, *p* = 0.01) (Figure 2). Apelin levels were not significantly higher in the group with HbA1c ≥ 7.0% (7.25 ng/mL, 95% CI = 6.37–8.30 ng/mL) than in the group with HbA1c < 6.9% (7.63 ng/mL; 95% CI = 6.60–8.72 ng/mL, *p* = 0.20). The levels of myostatin (13.80 ng/mL, 95% CI = 10.40–16.80 ng/mL and 12.50 ng/mL, 95% CI = 9.90–15.40 ng/mL, *p* = 0.66) and adropin (247.80 pg/mL, 95% CI = 203.50–296.70 pg/mL and 215.60 pg/mL, 95% CI = 188.20–257.40 pg/mL, *p* = 0.21) exhibited strict similarities amongst patients from both groups.

### 3.3. Spearman’s Correlation between Circulating Levels of Myokines and Other Parameters

We found positive correlations between irisin levels and LVEF (r = 0.37; *p* = 0.001), and an inverse correlation with NT-proBNP levels (r = −0.36; *p* = 0.001), NYHA class (r = −0.32; *p* = 0.001), the HOMA index (r = −0.31; *p* = 0.001), LVMMI (r = −0.33; *p* = 0.001), LAVI (r = −0.32; *p* = 0.001) and E/e’ (r = −0.30; *p* = 0.001), but not with BMI. Apelin levels correlated positively with NT-proBNP levels (r = 0.32; *p* = 0.001), HOMA index (r = 0.30; *p* = 0.001), LVMMI (r = 0.30; *p* = 0.001), NYHA class (r = 0.27; *p* = 0.01), LAVI (r = 0.30; *p* = 0.001) and E/e’ (r = 0.26; *p* = 0.001), and negatively with BMI (r =−0.31; *p* = 0.012). Myostatin levels were positively associated with HFrEF (r = 0.28; *p* = 0.001) and LAVI (r = 0.30; *p* = 0.001), and negatively associated with LVEF (r = −0.34; *p* = 0.001). Adropin levels were found to be significantly correlated with HfrEF (r = 0.34; *p* = 0.001), NT-proBNP levels (r = 0.36; *p* = 0.001), LAVI (r = 0.32; *p* = 0.001), BMI (r = 0.29, *p* = 0.01), NYHA class (r = 0.30, *p* = 0.012) and eGFR (r = −0.31; *p* = 0.001), but not associated with HOMA index. We did not observe significant correlations between the levels of myokines. However, there were no significant correlations between myokine levels and concomitant medications.

### 3.4. The Predictors of Poor Glycemic Control in T2DM Patients with HF: The Univariate and Multivariate Linear Regression

We identified several predictors of poor glycemic control in the study using a univariate linear regression model (Table 2). We established that HFrEF (OR = 1.04; 95% CI = 1.02–1.07, *p* = 0.042), LVEF (OR = 1.03; 95% CI = 1.01–1.05, *p* = 0.048), LAVI (OR = 1.05; 95% CI = 1.03–1.09, *p* = 0.044), NT-proBNP levels (OR = 1.07; 95% CI = 1.03–1.12, *p* = 0.01), irisin levels (OR = 1.09; 95% CI = 1.05–1.16, *p* = 0.001), apelin levels (OR = 1.07; 95% CI = 1.02–1.11, *p* = 0.024) and myostatin levels (OR = 1.04; 95% CI = 1.01–1.06, *p* = 0.044) had predictive value for patients with HbA1c ≥ 7.0%. Multivariate linear regression suggested that levels of NT-proBNP (OR = 1.07; 95% CI = 1.02–1.10, *p* = 0.04) and irisin (OR = 1.09; 95% CI = 1.04–1.17, *p* = 0.001) remained independent predictors for the dependent variable.

### 3.5. Comparison of the Models

To compare the two models, we used the area under the curve estimate, which showed that the predictive ability of irisin was better compared to that of NT-proBNP (*p* = 0.001). Nevertheless, irisin enhanced risk differentiation by increasing the prognostic impact of the dependent variable independently of NT-proBNP (Table 3). Moreover, the combination of the two biomarkers (NT-proBNP + irisin) did not improve the discriminative power of irisin alone (AUC = 0.82; 95% CI = 0.75–0.90 vs. AUC = 0.81; 95% CI = 0.73–0.89, *p* = 0.86).

## 4. Discussion

The results of the study confirmed our initial working hypotheses that poor glycemic control in T2DM patients with HF may lead to worsened cardiac performance through an altered myokine signature. We found that patients with HbA1c ≥ 7.0% had lower levels of irisin than those with HbA1c < 6.9%, whereas other myokines (apelin, myostatin and adropin) did not exhibit significant differences in their concentrations in relation to glucose control. However, we confirmed that irisin levels correlated positively with LVEF and negatively with NT-proBNP levels, NYHA class, the HOMA index, LVMMI, LAVI and E/e’, but not with BMI and levels of other myokines. Moreover, the predictive ability of lower levels of irisin regarding poor glycemic control was higher than that of NT-proBNP and had value independent from other parameters including several clinical characteristics (NYHA class III vs. NYHA class I/II), the presence of HFrEF or AF, LVEF, diastolic filling abnormalities and other biomarkers (the HOMA index).

The strength of these results was that the majority of the patients included in the study received conventional HF therapies, including SGLT2 inhibitors, so that these findings may open perspectives for predicting treatment response in HF patients with T2DM with any levels of NT-proBNP. Indeed, previous clinical studies have clearly shown that patients with any HF phenotype have an elevated risk of mortality and hospitalization beyond that estimated by levels of natriuretic peptide [8] and that some antidiabetic agents, such as glucagon-like peptide-1 receptor agonist and SGLT2 inhibitors, improved the clinical course of HF even without significant changes in levels of NPs amongst patients with T2DM [27,28,29,30].

In this context, circulating myokines seem to show their predictive potency for adverse cardiac remodeling, new HF and progression of known HF regardless of NPs [31]. Previous studies have revealed that irisin, apelin and adropin adaptively mediate metabolic regulations of the myocardium, skeletal muscles and vascular and adipose tissue. These myokines also demonstrated a beneficial protective effect on the heart by attenuating LVH and fibrosis of extracellular matrix by improving cardiomyocyte viability and by reducing systemic and local (microvasculature and adipose tissue) inflammatory responses, while myostatin contributed to maladaptive changes in cardiac and skeletal muscle structure and metabolism [32,33,34,35]. The dynamic changes of irisin and apelin are considered to be predictive factors for impaired insulin sensitivity in peripheral tissues, mainly skeletal muscles [34,35]. However, there was no strong evidence to support the fact that circulating levels of these myokines could be used instead of conventional parameters of glycemic control, such as fasting glucose and HbA1c [9,10,33,35].

Circulating levels of irisin and apelin were found to be significantly decreased in chronic HF patients depending on severity of the disease, whereas elevated levels of adropin and myostatin have been frequently noticed in acute HF patients and especially in acutely decompensated HFrEF patients [36,37]. Perhaps several clinical features of HF patients including fatigue, altered physical tolerance, myopathy and cardiac cachexia may be regulated by an altered myokine profile [38]. Moreover, these myokines were important predictive factors for shortening survival in patients with any HF phenotype [39,40,41]. It has been suggested that the metabolic effects of the myokines that contribute to the myocardial–skeletal muscle axis should be regarded as a critical element of T2DM-induced impairment of cardiac function.

We first established that the levels of irisin, but not apelin, adropin and myostatin differed from those of T2DM patients with chronic HF depending on glycemic control. It is possible that significant differences in other myokines might appear due to fluid retention, HF decompensation, a more advanced stage of HF or the prevalence of HFrEF in the study population. However, we hypothesized that poor glycemic control is able to intervene in worsening cardiac structure and function through an imbalance between adaptive and maladaptive myokines. Indeed, there was a correspondence between altered irisin levels and abnormalities in echocardiographic parameters describing LV diastolic filling, which were more altered in the group with HbA1c ≥ 7.0%. Other investigators have also reported that circulating irisin levels correlate with metabolic and inflammatory parameters and the clinical course of HF in HFpEF and HFrEF patients, regardless of HOMA index [42,43]. However, data on the adaptive role of irisin in HF patients with T2DM are quite restricted.

Overall, irisin is an adaptive myokine that is considered to be a physiological antagonist of renin–angiotensin–aldosterone system (RAAS) and sympathoadrenal system (SAS). Hyperactivity in both systems promotes a majority of the hemodynamic, metabolic, inflammatory and proliferative responses in HF and T2DM, contributing to the overlap between the pathogenetic mechanisms of these conditions [44]. One of most common molecular mechanisms by which irisin counteracts the activity of RAAS and SAS is its ability to booster the activity of AMP kinase, but not Akt and MAP kinase, thereby protecting cardiomyocytes from hypertrophy induced by angiotensin II or phenylephrine, and suppressing fibrosis, gluconeogenesis, lipolysis, oxidative stress and inflammation [45]. The next mechanism is irisin-induced protective autophagy, which not only attenuates cardiac hypertrophy [46], but also improves the tolerability of cardiac myocytes against apoptosis and necrosis due to hypoxia and ischemia [47]. Moreover, irisin is able to regulate the metabolism of skeletal muscles in an autocrine manner [48], thereby reducing resistance to insulin, which plays a central role in altered reparative tissue potency in T2DM as well as in HF [49]. The final, but not least important, molecular mechanism for the beneficial impact of irisin on the myocardium, skeletal muscles and vasculature is its ability to regulate miRNA-19b expression, thereby reactivating the AKT/mTOR signaling pathway, ensuring the suppression of H_2_O_2_ production and preventing mitochondrial dysfunction [50]. All these underlying molecular mechanisms seem to partially explain the findings of our study, but the main reason for the difference in irisin concentrations between HF patients with and without poor glycemic control remains unclear. This requires more investigation in the future.

Future research may highlight the role of improved glycemic control in altering HF phenotypes and reducing the risks of HF-related outcomes and mortality in the HF population with T2DM. However, the widespread implementation of SGLT2 inhibitors in patients with HFrEF and also in patients with HFpEF regardless of T2DM suggest that new biomarkers such irisin are considered useful in the context of improving the discriminative power of current models, even if NT-proBNP levels remain respectively lower. Perhaps myokine-based models may be useful in practice to clearly evaluate the risk of newly diagnosed T2DM in HF patient population.

## 5. Study Limitations

The study has several limitations. First of all, it is a retrospective study. The next limitation is related to the lack of continuous biomarker monitoring in T2DM patients, such that it is plausible that poor glycemic control impacted the transition to HF phenotypes. The last but not least important limitation was the lack of data on improvements in glycemic control in relation to the dynamics of circulating biomarkers and their ability to predict clinical outcomes as a function of glycemic control. Despite the fact that irisin is considered to be the weaker indicator of glucose control when compared with the canonic HbA1c or fasting glucose, more investigations are required to clarify this notion. Our study was not designed to elucidate the issue. We believe that these limitations are not critical for the interpretation of the results of the study and that they do not restrict the extrapolation of our findings to other populations of HF patients with T2DM.

## 6. Conclusions

We found that decreased levels of irisin were associated with poor glycemic control in T2DM patients with HF, regardless of clinical conditions and other biomarkers. We consider this finding to be of interest for the discovery of new predictive models, which could allow one to determine the risk of HF patients with any concentrations of natriuretic peptide, independent of HF phenotype and the presence of several comorbidities.

## Figures and Tables

**Table 1:** Basic demographic, clinical, and hemodynamic characteristics, biochemistry biomarkers and concomitant medications of patients in the study population.

Variables	Entire T2DM Patient Cohort with HF (*n* = 314)	Patients with HbA1c < 6.9% (*n* = 183)	Patients with HbA1c ≥ 7.0% (*n* = 131)	*p*-Value
Age, year	52 (40–62)	51 (41–62)	52 (40–60)	0.82
Male, *n* (%)	205 (65.3)	118 (64.5)	87 (66.4)	0.86
Dyslipidemia, *n* (%)	268 (85.3)	152 (83.1)	116 (88.5)	0.82
Hypertension, *n* (%)	267 (85.0)	158 (86.3)	109 (83.2)	0.88
Stable CAD, *n* (%)	135 (43.0)	74 (40.4)	61 (45.6)	0.62
Paroxysmal/persistent AF, *n* (%)	72 (22.9)	35 (19.1)	37 (28.2)	0.01
Smoking, *n* (%)	157 (50.0)	89 (48.6)	68 (51.9)	0.58
Abdominal obesity, *n* (%)	158 (50.3)	84 (45.9)	74 (56.5)	0.046
Microalbuminuria, *n* (%)	109 (34.7)	56 (30.6)	53 (40.5)	0.042
LVH, *n* (%)	266 (84.7)	144 (78.7)	122 (93.1)	0.04
BMI, kg/m^2^	26.1 ± 1.5	25.8 ± 2.1	26.7 ± 2.0	0.86
Waist circumference, sm	86.5 ± 2.0	85.6 ± 2.9	87.2 ± 2.2	0.84
WHR, units	0.86 ± 0.04	0.86 ± 0.03	0.88 ± 0.02	0.82
HFpEF, *n* (%)	124 (39.5)	72 (39.3)	52 (39.7)	0.84
HFmrEF, *n* (%)	105 (33.4)	58 (31.7)	47 (35.9)	0.78
HFrEF, *n* (%)	85 (27.1)	53 (29.0)	32 (24.4)	0.86
I/II/III NYHA class, *n*	46/165/103	30/103/50	16/62/53	0.24
SBP, mm Hg	131 ± 5	132 ± 5	130 ± 4	0.92
DBP, mm Hg	79 ± 4	80 ± 4	78 ± 3	0.90
LVEDV, mL	161 ± 7	154 ± 9	168 ± 6	0.72
LVESV, mL	84 ± 4	74 ± 7	86 ± 2	0.73
LVEF, %	49 ± 5	51 ± 6	48 ± 4	0.44
LVMMI, g/m^2^	164 ± 5.60	151 ± 6.12	172 ± 4.42	0.044
LAVI, mL/m^2^	46 ± 6	39 ± 8	54 ± 5	0.046
E/e’, unit	14.5 ± 0.8	13.9 ± 0.5	15.2 ± 0.7	0.01
eGFR, mL/min/1.73 m^2^	81 ± 5.1	83 ± 6.0	79 ± 5.6	0.82
HOMA-IR, units	9.74 ± 3.1	7.65 ± 3.7	11.40 ± 2.4	0.05
NT-proBNP, pmol/mL	2855 (1430–4140)	2718 (1380–3720)	2982 (1640–4527)	0.12
Fasting glucose, mmol/L	6.65 ± 1.93	5.84 ± 1.22	7.34 ± 1.38	0.01
Creatinine, µmol/L	110.4 ± 14.2	108.8 ± 12.0	114.2 ± 10.3	0.28
HbA1c, %	7.01 ± 0.5	6.47 ± 0.3	7.42 ± 0.4	0.04
TC, mmol/L	6.53 ± 0.06	6.41 ± 0.05	6.62 ± 0.07	0.88
HDL-C, mmol/L	0.94 ± 0.19	0.95 ± 0.21	0.93 ± 0.18	0.84
LDL-C, mmol/L	4.50 ± 0.15	4.43 ± 0.20	4.51 ± 0.16	0.88
TG, mmol/L	2.27 ± 0.05	2.26 ± 0.04	2.31 ± 0.03	0.88
SGLT2i, *n* (%)	293 (93.3)	171 (93.4)	122 (93.1)	0.90
ACEIs/ARBs/ARNI, *n* (%)	274 (87.3)	158 (86.3)	116 (88.5)	0.89
MRA, *n* (%)	85 (27.1)	53 (29.0)	32 (24.4)	0.86
Statins, *n* (%)	268 (85.3)	152 (83.1)	116 (88.5)	0.82
Beta-blockers, *n* (%)	292 (93.0)	174 (95.0)	118 (90.1)	0.22
Ivabradin, *n* (%)	37 (11.8)	23 (12.6)	14 (10.7)	0.68

Notes: data are given as mean ± SD and median (interquartile range). Variables were compared with the Tukey test. Abbreviations: ACEIs, angiotensin-converting enzyme inhibitors; ARBs, angiotensin-II receptor blockers; ARNI, angiotensin receptor/neprilysin inhibitor; BMI, body mass index; CAD, coronary artery disease; DBP, diastolic blood pressure; E/e’, early diastolic blood filling to longitudinal strain ratio; GFR, glomerular filtration rate; LDL-C, low-density lipoprotein cholesterol; HbA1c, glycosylated hemoglobin; HDL-C, high-density lipoprotein cholesterol; HF, heart failure; HOMA-IR, Homeostatic Assessment Model of Insulin Resistance; HFpEF, heart failure with preserved ejection fraction; HFmrEF, heart failure with mildly reduced ejection fraction; HFrEF, heart failure with reduced ejection fraction; NT-proBNP, N-terminal brain natriuretic pro-peptide; LVEDV, left ventricular end-diastolic volume; LVESV, left ventricular end-systolic volume; LVEF, left ventricular ejection fraction; LVH, left ventricle hypertrophy; LAVI; left atrial volume index; MRA, mineralocorticoid receptor antagonists; SBP, systolic blood pressure; SGLT2i, sodium–glucose cotransporter-2 inhibitor; T2DM, type 2 diabetes mellitus; TG, triglycerides; TC, total cholesterol; WHR, waist-to-hip ratio.

**Table 2:** Predictors of poor glycemic control in the study population. The results of the univariate and multivariate linear regression analyses adjusted to HOMA index and BMI.

	Dependent Variable: HbA1c ≥ 7.0%			
Variables	Univariate Linear Regression		Multivariate Linear Regression	
	OR (95% CI)	*p*-value	OR (95% CI)	*p*-value
III NYHA class vs. I/II NYHA class	1.03 (1.00–1.07)	0.050	-	
AF versus sinus rhythm	1.08 (0.93–1.17)	0.82	-	
HFrEF vs. HFpEF/HFmrEF	1.04 (1.02–1.07)	0.042	1.05 (1.01–1.09)	0.050
LVEF	1.03 (1.01–1.05)	0.048	1.02 (1.00–1.05)	0.28
LAVI	1.05 (1.03–1.09)	0.044	1.03 (1.00–1.07)	0.050
E/e’	1.02 (0.98–1.05)	0.86	-	
NT-proBNP	1.07 (1.03–1.12)	0.01	1.07 (1.02–1.10)	0.04
Irisin	1.09 (1.05–1.16)	0.001	1.09 (1.04–1.17)	0.001
Apelin	1.07 (1.02–1.11)	0.024	1.05 (1.00–1.11)	0.050
Myostatin	1.04 (1.01–1.06)	0.044	1.02 (1.00–1.04)	0.052
Adropin	1.07 (0.99–1.12)	0.850	-	

Abbreviations: AF, atrial fibrillation; OR, odds ratio; CI, confidence interval; E/e’, early diastolic blood filling to longitudinal strain ratio; LVEF, left ventricular ejection fraction; LAVI; left atrial volume index; NT-proBNP, N-terminal brain natriuretic pro-peptide; HFrEF, heart failure with reduced ejection fraction; NYHA, New York Heart Association.

**Table 3:** Comparison of the discriminative powers of NT-proBNP and irisin for predicting poor glycemic control.

Models	AUC		NRI		IDI	
	M (95% CI)	*p*-value	M (95% CI)	*p*-value	M (95% CI)	*p*-value
NT-proBNP	0.66 (0.60–0.74)	-	Reference	-	Reference	-
Irisin	0.81 (0.73–0.89)	0.001	0.34 (0.31–0.37)	0.01	0.45 (0.40–0.52)	0.01

Abbreviations: AUC, area under curve; CI, confidence interval; M, median; IDI, integrated discrimination indices; NRI, net reclassification improvement.

## Data Availability

Not applicable.
